# Case Report: Unveiling the unexpected: a rare case of adult-type rhabdomyoma in a 9-year-old boy

**DOI:** 10.3389/fped.2025.1451316

**Published:** 2025-02-03

**Authors:** Li Wang, Shan Li, Lingling Sun

**Affiliations:** ^1^ Department of Otolaryngology, Liaocheng People's Hospital, School of Medicine, Liaocheng University, Liaocheng, Shandong, China; ^2^ Department of Oncology, The Fifth People's Hospital of Jinan, Jinan, Shandong, China; ^3^ Department of Otolaryngology, Liaocheng People's Hospital, Liaocheng, Shandong, China

**Keywords:** adult-type rhabdomyoma, extracardiac rhabdomyomas, pharyngeal, child, case report

## Abstract

Rhabdomyoma is an exceedingly rare benign soft tissue tumor of skeletal muscle origin, classified into cardiac and extracardiac types based on location. Extracardiac rhabdomyoma further includes adult, genital, and fetal types depending on the degree of differentiation. Most patients are between 40 and 70 years old, with a mean age of 60 years and a male predominance. This case report presents a 9-year-old boy diagnosed with an oropharyngeal tumor, initially presenting with a 6-month history of a foreign body sensation in the throat, presenting as night-time snoring. Postoperative histological examination revealed adult rhabdomyoma, characterized by specific immunohistochemical and histologic traits including cytoplasmic positivity for muscle-specific Actin (MSA), Desmin, Myogenin, and MYOD1, large polygonal skeletal muscle cells and frequent extensive vacuolization. This report highlights the unusual age of presentation for this variant and underscores the need for heightened clinical awareness to ensure accurate diagnosis and effective management of such rare occurrences.

## Introduction

Rhabdomyomas are exceptionally rare benign soft tissue tumors originating from skeletal muscle cells (1), benign rhabdomyomas arise frequently in the head and neck (2), and constituting merely 2% of all skeletal muscle tumors (3). They are classified into cardiac and extracardiac types according to their location (4). Extracardiac rhabdomyomas are particularly rare (5, 6) and are further divided into fetal, juvenile, and adult subtypes based on histological features rather than the age of occurrence, although fetal rhabdomyoma typically presents in newborns and early childhood (1).

Adult rhabdomyomas predominantly affecting individuals between 40 and 70 years old, with a mean age of 60 years, and showing a male predominance (7). To date, the occurrence of adult-type rhabdomyoma in children's pharyngeal region has not been documented globally.

## Case description

**Figure 1:**
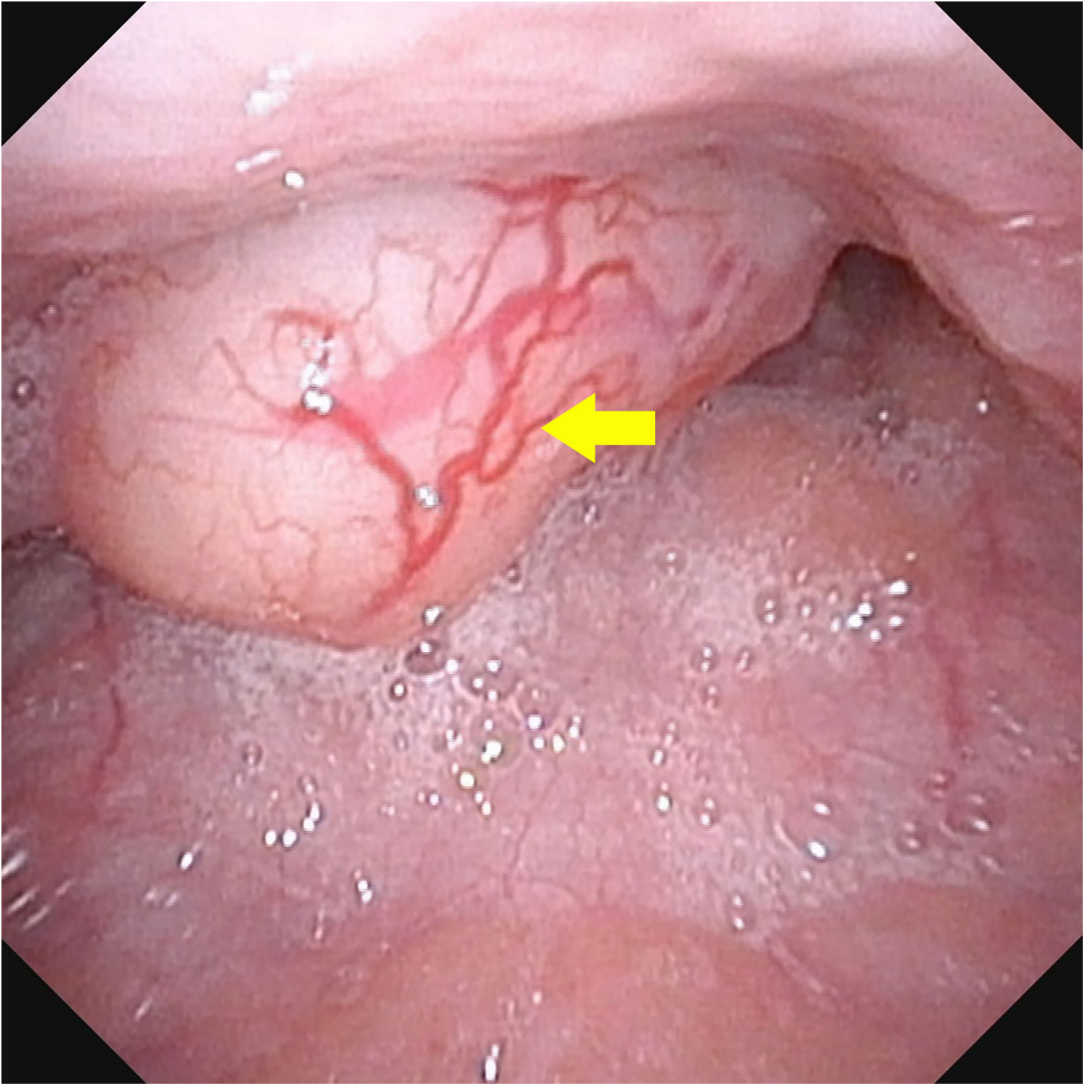
Fiberlaryngoscopic examination revealed a round mass with a pedicle in the pharynx, approximately 2 cm in diameter. The surface of the mass was smooth, with the pedicle rooted at the upper pole of the tonsil.

A 9-year-old boy was referred with a 6-month history of a foreign body sensation in the throat, presenting as night-time snoring. Fiberlaryngoscopic examination revealed a round mass with a pedicle in the pharynx, approximately 2 cm in diameter. The surface of the mass was smooth, with the pedicle rooted at the upper pole of the tonsil (Figure 1). The boy underwent a transoral excision of the oropharyngeal tumor.

**Figure 2:**
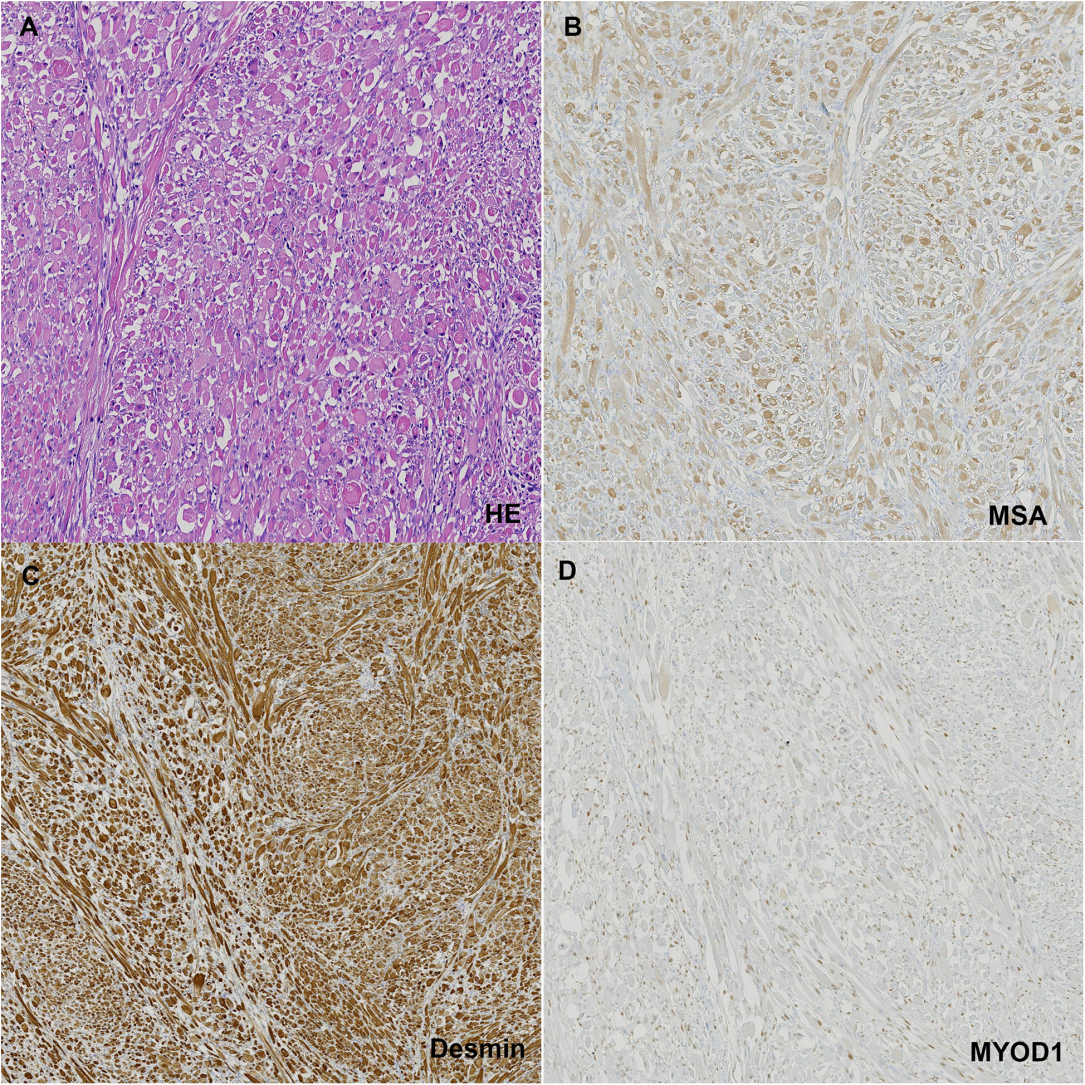
**(A)** HE staining of the tumor tissue; **(B)** positive staining with MSA; **(C)** positive staining with desmin; **(D)** positive staining with MYOD1.

Macroscopically, the mass measured 2.2 × 2.0 × 1.3 cm, was well-circumscribed, and on cut surface, appeared deep gray with a tough, braided texture. Microscopically, the cells exhibited abundant eosinophilic, granular cytoplasm with well-defined borders, large polygonal skeletal muscle cells and frequent extensive vacuolization. Immunohistochemical staining was positive for MSA, Desmin, MYOD1 (Figure 2) and Myogenin, leading to a final pathological diagnosis of adult rhabdomyoma.

The postoperative course was uneventful, and the boy was discharged five days after the intervention, with complete resolution of symptoms. He has been followed up for 18 months without any recurrence.

## Discussion

Extracardiac rhabdomyomas are exceedingly rare tumors, constituting less than 2% of neoplasms of striated muscle origin. Adult-type rhabdomyomas are rare benign tumors with a significantly lower incidence compared to their malignant counterpart, rhabdomyosarcoma (8). Adult rhabdomyomas predominantly affect the head and neck region, originating from the musculature of the third and fourth branchial arches. Most cases are solitary (70%) and typically occur in males over 50 years old (9). However, the occurrence of adult rhabdomyoma originating from a child's pharyngeal region is exceptionally rare. To our knowledge, this is the first reported case of an adult rhabdomyoma in the pharyngeal region of child.

The signs and symptoms at the time of presentation depend on the tumor's location. In our case, the boy experienced a sensation of a foreign body in the throat and presented with night-time snoring for six months. Reported symptoms in the literature include hearing loss, a mass in the submandibular triangle, swelling in the nasopharynx, hemoptysis, and obstructive sleep apnea, though pure dysphagia is rare (7, 10, 11).

Current diagnostic imaging modalities, including ultrasound, CT, and MRI, have not proven to be highly specific for the diagnosis of adult rhabdomyoma (8). In our case, there was an objective image of a mass with a pedicle in the pharynx, prompting us to perform direct excision. It is importance to combine imaging with histopathological confirmation, especially in pediatric patients where rare tumors like rhabdomyomas are less frequently considered. While imaging is useful for localization and surgical planning, histopathology remains the definitive diagnostic method. The gold standard treatment is surgery. Despite adult rhabdomyomas being benign, up to 42% may recur due to incomplete resection (12). Moreover, residual tumors may have malignant potential (13).

The microscopic appearance of adult rhabdomyomas is relatively consistent, characterized by polygonal, closely packed cells with prominent nuclei located centrally or peripherally. The cytoplasm is eosinophilic, granular, and shows extensive vacuolization in some cells. In our case, these features were observed. Immunohistochemical diagnosis features of rhabdomyoma include cytoplasmic positivity for MSA, Desmin, myoglobin, and MYOD1 (14).

## Conclusion

Adult rhabdomyoma in children is an exceptionally rare occurrence and presents a diagnostic challenge due to its atypical manifestation in pediatric head, oral cavity, and neck tumors. Surgical resection is the treatment of choice, and careful long-term follow-up is essential. This case highlights the importance of considering adult rhabdomyoma in the differential diagnosis of pediatric pharyngeal masses, despite its rarity in this age group. Monitoring for recurrence is crucial in our clinical center to ensure optimal management and outcomes for this patient.

## Data Availability

The original contributions presented in the study are included in the article/Supplementary Material, further inquiries can be directed to the corresponding author.
